# Synthesis, structural and spectroscopic characterization of defect-rich forsterite as a representative phase of Martian regolith

**DOI:** 10.1107/S2052252524009722

**Published:** 2024-10-28

**Authors:** Muchammad Izzuddin Jundullah Hanafi, Lorenzo Bastonero, Mohammad Mangir Murshed, Lars Robben, Wilke Dononelli, Andrea Kirsch, Nicola Marzari, Thorsten M. Gesing

**Affiliations:** a University of Bremen Institute of Inorganic Chemistry and Crystallography Leobener Straße 7 D-28359Bremen Germany; b University of Bremen MAPEX Center for Materials and Processes Bibliothekstraße 1 D-28359Bremen Germany; c Bremen Center for Computational Materials Science and Hybrid Materials Interfaces Group, Am Fallturm 1, D-28359Bremen, Germany; d University of Copenhagen Department of Chemistry and Nanoscience Center Universitetsparken 5 Copenhagen2100 Denmark; Formby, Liverpool, United Kingdom

**Keywords:** Martian forsterite, crystal structures, defects, DFT–PDF, Raman spectroscopy, X-ray powder diffraction, ball milling

## Abstract

Regolith draws intensive research attention because of its importance as the basis for fabricating materials for future human space exploration. Martian regolith is predicted to consist of defect-rich crystal structures due to long-term space weathering. The present report focuses on the structural differences between defect-rich and defect-poor forsterite (Mg_2_SiO_4_) – one of the major phases in Martian regolith. In this work, forsterites were synthesized using reverse strike co-precipitation and high-energy ball milling (BM). Subsequent post-processing was also carried out using BM to enhance the defects. The crystal structures of the samples were characterized by X-ray powder diffraction and total scattering using Cu and synchrotron radiation followed by Rietveld refinement and pair distribution function (PDF) analysis, respectively. The structural models were deduced by density functional theory assisted PDF refinements, describing both long-range and short-range order caused by defects. The Raman spectral features of the synthetic forsterites complement the *ab initio* simulation for an in-depth understanding of the associated structural defects.

## Abstract 2

Ball milling of forsterite (Mg_2_SiO_4_) was carried out to mimic mechanical weathering processes on Mars. The defective forsterite structure models, capable of describing both long-range and short-range order, are deduced by density functional theory assisted pair distribution function analysis.

## 1. Introduction

In recent years, knowledge about Martian regolith has drastically increased due to the availability of *in-situ*X-ray diffraction data from the Mars Science Laboratory (MSL) on the Rover Curiosity (Bish *et al.*, 2013 ▸; Achilles *et al.*, 2017 ▸; Vaniman *et al.*, 2014 ▸). The analysis of these diffraction data estimated an amorphous fraction of approximately 28 to 45 wt% in the Martian regolith (Certini *et al.*, 2020 ▸; Achilles *et al.*, 2017 ▸; Vaniman *et al.*, 2014 ▸; Demidov *et al.*, 2015 ▸; Bish *et al.*, 2013 ▸). A notable amorphous phase content in Martian regolith indicates significant space weathering due to extreme environmental conditions (Certini *et al.*, 2020 ▸). Space weathering is the alteration of exposed surfaces via their interaction with the space environment (Bennett *et al.*, 2013 ▸). It is a combination of mechanical weathering caused, for example, by meteorite impacts as well as radiation weathering from high-energy solar wind radiation. The former process can be simulated by ball milling (BM) of terrestrial materials in the laboratory (Yu *et al.*, 2022 ▸).

Olivine-type forsterite (Mg_2_SiO_4_) is one of the major phases found in the crystalline part of Martian regolith (Bish *et al.*, 2013 ▸; Achilles *et al.*, 2017 ▸). Bish *et al.* (2013 ▸) reported it to be comprised of approximately 22.4 wt% (Mg_0.62_Fe_0.38_)_2_SiO_4_, known as forsteritic olivine. The term can be understood from the forsterite–fayalite [(Mg_1−*x*_Fe*_x_*)_2_SiO_4_] solid solution due to higher Mg content compared with Fe, which was found in Martian soil from the Rocknest Aeolian bedform in the Gale crater. Similarly, Achilles *et al.* (2017 ▸) found approximately 25.8 wt% forsteritic olivine [(Mg_0.56_Fe_0.44_)_2_SiO_4_] in Martian soil of the Namib dune named Gobabeb.

For future use of regolith as a basis to fabricate metals or building materials for human space explorations, a precise analysis of the different defects present in forsterites is of crucial importance. The defect-rich forsterite is expected to have lower formation energy, hence may be desirable for more efficient processing of fabrication in space.

**Figure 1:**
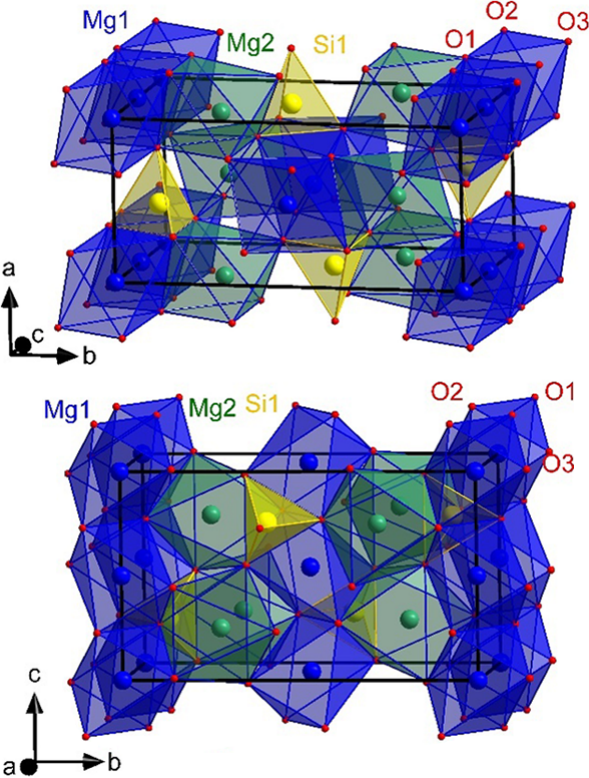
Crystal structure of Mg_2_SiO_4_ (forsterite).

Forsterite belongs to planetary and terrestrial rock-forming minerals (Liu *et al.*, 2022 ▸; Váci *et al.*, 2020 ▸) and is known for its capability of catalyzing reactions in interstellar dust (Campisi *et al.*, 2024 ▸). Forsterite is the magnesium endmember of the olivine solid solution (Mg_1−*x*_Fe*_x_*)_2_SiO_4_ (Jundullah Hanafi *et al.*, 2024 ▸; Rösler, 1991 ▸) and crystallizes in the orthorhombic space group *Pbnm* (Fujino *et al.*, 1981 ▸; Müller-Sommer *et al.*, 1997 ▸; Lager *et al.*, 1981 ▸). The structure (Fig. 1 ▸) consists of 1D octahedral chains running parallel to the crystallographic *c* axis, comparable to those found in the mullite-type phase (Angel & Prewitt, 1986 ▸; Bowen *et al.*, 1924 ▸; Cong *et al.*, 2010 ▸; Fischer *et al.*, 2009 ▸; Gogolin *et al.*, 2020 ▸). In the mullite-type phase, these octahedral chains are bridged by double tetrahedra or other double units in the *ab* direction (Murshed *et al.*, 2012 ▸; Schneider *et al.*, 2012 ▸), whereas in olivine, single (SiO_4_)^4−^ tetrahedra link the octahedral chains in the *a* direction, where the tetrahedrally coordinated oxygen atoms are shared by three octahedrally coordinated cations (Zampiva *et al.*, 2017 ▸). The respective link in the *b* direction is realized by two non-equivalent Mg octahedral sites: the first site (*M*1, chain octahedra) has inversion symmetry, while the other site (*M*2, linking octahedra) possesses mirror symmetry (Yang *et al.*, 2006 ▸). Both sites can be occupied by various cations, forming either rich solid solutions or other endmembers such as fayalite [Fe_2_SiO_4_ (Hanke, 1965 ▸; Kudoh & Takeda, 1986 ▸; Hazen, 1977 ▸)], tephroite [Mn_2_SiO_4_ (Fujino *et al.*, 1981 ▸)], larnite [Ca_2_SiO_4_ (Czaya, 1971 ▸)], liebenbergite [Ni_2_SiO_4_ (Della Giusta *et al.*, 1990 ▸; Lager *et al.*, 1981 ▸)] and cobalt olivine [Co_2_SiO_4_ (Morimoto *et al.*, 1974 ▸; Müller-Sommer *et al.*, 1997 ▸)], which enable a wider spectrum of elements extractable from a regolith matrix.

Forsterite can be synthesized through a variety of synthesis methods including the solution combustion technique (Naik *et al.*, 2015 ▸; Mondal *et al.*, 2016 ▸; Prashantha *et al.*, 2011 ▸), the sol–gel method (Ni *et al.*, 2007 ▸), mechanical activation followed by heat treatment (Fathi & Kharaziha, 2008 ▸, 2009 ▸; Tavangarian & Emadi, 2010 ▸) and reverse strike co-precipitation (RSC) (Zampiva *et al.*, 2017 ▸). Despite many studies on the synthesis yielding pure forsterite, to the best of our knowledge there are no reports on mechanically induced defect-rich forsterites and their associated crystal structures. Defects are commonly defined as a considerable extent of irregularities in the crystal structure (Wagner, 1977 ▸), for example, vacancies or dislocated atoms (see Fig. S1 of the supporting information). In an X-ray diffraction pattern, typical strain broadening and lower absolute intensities are expected for defect-rich crystallites (Ungár, 2004 ▸, Chauhan & Chauhan, 2014 ▸), often accompanied by reflection broadening due to crystallite size effects (Scherrer, 1918 ▸; Gesing & Robben, 2024 ▸). Similarly, broadening of Raman peaks suggests disordered structures in defect-rich materials (Demtröder, 2008 ▸; Gouadec & Colomban, 2007 ▸). In addition to Rietveld analysis of reciprocal-space X-ray powder diffraction (XRPD) data, real-space investigations of defects and local structures are widely performed by pair distribution function (PDF) analysis (Bini *et al.*, 2012 ▸; Malavasi *et al.*, 2011 ▸; Proffen *et al.*, 2003 ▸).

The present work focuses on the synthesis and characterization of forsterite and its mechanical post-treatment to induce various defect concentrations. The derived defect-structure model can serve as a structural representative for the analysis of Martian regolith. To achieve this objective, Mg_2_SiO_4_ samples were first synthesized by two different routes: the RSC method and mechanical activation using high-energy BM with subsequent calcination. In a second step, mechanical post-treatment was performed to obtain defect-rich forsterite. We present a detailed comparison of structural features between the samples using Raman, XRPD and X-ray total scattering techniques. In addition, density functional theory (DFT) is used to optimize defect-rich structures and compare their thermodynamic stability. Finally, the DFT-supported PDF analysis (DFT–PDF) is used to refine the defective structural models.

## 2. Materials and methods

### 2.1. Synthesis of defect-poor forsterite

#### 2.1.1. Reverse strike co-precipitation synthesis

Magnesium nitrate hexahydrate [Mg(NO_3_)_2_·6H_2_O, 99.9 %] and tetra­ethyl orthosilicate (TEOS, 98 %) were purchased from VWR Chemicals and used as received. Similar to a typical RSC synthesis (Zampiva *et al.*, 2017 ▸), stoichiometric amounts of Mg(NO_3_)_2_·6H_2_O and TEOS were dissolved in a solution of 40 ml of ethanol and 3 ml of HNO_3_ (≥65 %) under magnetic stirring for 1 h. The precursor solution was slowly dripped into 50 ml of 25 % NH_4_OH under continuous stirring. The base solution formed a colloid while the precursor solution was being dripped, forming a white precipitate. Simultaneously, several drops of concentrated NH_4_OH were added to maintain pH > 8. Thereafter, the precipitate was centrifuged at 60 Hz for 5 min. The supernatant was removed, and ethanol was used to wash the precipitated powder. The centrifugation was repeated three times. Finally, the precipitated solid was placed in a furnace at 473 K for 16 h. The resulting solid was ground in a mortar and calcined at 1373 K for 1 h after reaching the temperature with heating and cooling rates of 15 and 5 K min^−1^, respectively. The powder attained is designated RFO (RSC synthesis forsterite).

#### 2.1.2. Ball milling synthesis

MgO and amorphous SiO_2_ powders were used as starting materials. MgO (>97 %) was purchased from Merck and used as received. Amorphous SiO_2_ was obtained from hydrolysis of TEOS (De *et al.*, 2000 ▸). Forsterite was synthesized by mechanical activation with a high-energy ball mill (Emax-type, RETSCH GmbH). Stoichiometric amounts of the binary oxides were mixed together with 60 g tungsten carbide (WC) balls (2 mm diameter) and placed in a WC grinding jar. The ball-to-powder weight ratio was set to 30:1. The powder was milled for 3 h with different rotational frequencies (7, 12.5 and 15 Hz). Finally, the milled powder was collected from the grinding jar and heated in a corundum crucible at 1373 K at a heating rate of 15 K min^−1^. After a reaction period of 1 h, the powder was cooled to room temperature at a cooling rate of 5 K min^−1^. The Mg_2_SiO_4_ pristine forsterite (PFO) powder attained was ground and further used for characterization.

### 2.2. Synthesis of defect-rich and healed forsterite

Defect-rich forsterite was prepared by crushing PFO by BM. PFO (1 g) was placed into the grinding jar with 20 g WC balls. The powder was milled at 15 Hz for 1 h. Thereafter, one half of the milled powder was kept separately and labeled as crushed forsterite (CFO). The other half was calcined at 1373 K for 1 h under air to heal the introduced defects, hence labeled healed forsterite (HFO).

### 2.3. X-ray powder diffraction

XRPD data collection was carried out on a Bruker D8 Discover diffractometer using Cu *K*α_1,2_ radiation [λ_*K*α_1__ = 154.05929(5) pm, λ_*K*α_2__ = 154.4414(2) pm] in Bragg–Brentano geometry. Data were collected under ambient conditions from 5 to 85° 2θ with a step width of 0.0149° 2θ and a measurement time of 0.3 s per step using a multi-strip LynxEye XE-T detector. XRPD data Rietveld refinements were carried out using *TOPAS* (version 6.0). During the Rietveld refinements, the background, sample displacement, cell metrics, atomic positions and profile parameters were optimized. The amorphous fraction of the samples was quantified from the degree of crystallinity (DC) as implemented in the *TOPAS* software. For these calculations it is assumed that the average scattering power of the crystalline fraction of the sample is identical to the scattering power of the X-ray amorphous fraction. The latter could either consist of glassy or quantum-crystalline contributions (Gesing *et al.*, 2022 ▸). Using the fundamental parameter approach (Cline *et al.*, 2010 ▸), the apparent average crystallite size (ACS) was calculated from all observed X-ray reflections, which is described as *L*_Vol_(*IB*) by the *TOPAS* suite. *L*_Vol_(*IB*) refers to the volume-weighted mean of the coherently diffracting domain size using the integral breadth for the description of the reflection profile. The respective pseudo-Voigt profile function was deconvoluted into Gaussian and Lorentzian components, describing the ACS and the microstrain (ɛ_0_), respectively. To validate these data and to receive information about the crystallite size distribution (CSD), an EnvACS analysis (Gesing & Robben, 2024 ▸) was performed. For this, data were collected on a Bruker D8 Advance diffractometer using Cu *K*α_1_ radiation [λ_*K*α_1__ = 154.05929(5) pm] in Bragg–Brentano geometry. Data were collected under ambient conditions from 10 to 135° 2θ with a step width of 0.01449° 2θ and a measurement time of 4.8 s per step using a multi-strip LynxEye XE detector. The information deduced during these (classical) Rietveld refinements are, with the exception of the DC, based on the appearance of the Bragg reflections and an ideal arrangement of atoms in the unit cell only (Rietveld, 1969 ▸). To distinguish these calculations from those using total scattering data, we use the expression Bragg–Rietveld for the classical method. The *R*_wp_ given is the weighted profile *R* factor (residual) of the Bragg–Rietveld refinement. To distinguish the *R*_wp_ of Bragg–Rietveld and the *R*_wp_ of PDF refinements, the latter is denoted *R*_PDF_.

### 2.4. Raman spectroscopy

Raman spectra were recorded on a LabRam ARAMIS (Horiba Jobin – Yvon) Micro-Raman spectrometer equipped with a green laser (λ_ex_ = 532 nm and < 20 mW power). A 50× objective (Olympus) with a numerical aperture of 0.75 provides a focus spot of 865 nm diameter when closing the confocal hole to 200 µm. Each spectrum ranges between 100 and 1200 cm^−1^ with a spectral resolution of approximately 1.2 cm^−1^ using a grating of 1800 grooves mm^−1^ and a thermoelectrically cooled CCD detector (Synapse, 1024 × 256 pixels).

### 2.5. Theoretical Raman calculations

The theoretical Raman spectra calculations were carried out using the *aiida-vibroscopy* package (Bastonero & Marzari, 2024 ▸) which exploits the finite displacements and finite field approach (Souza *et al.*, 2002 ▸; Umari & Pasquarello, 2002 ▸), and the *AiiDA* infrastructure (Huber *et al.*, 2020 ▸; Uhrin *et al.*, 2021 ▸) to automate the submission of the simulations and the storage of all the data in a reproducible format. The first-order spectrum was calculated in the non-resonant regime using the Placzek approximation. The peak positions associated with the phonon modes were computed in the harmonic approximation via small displacements of the atomic positions (Togo, 2023 ▸; Togo *et al.*, 2023 ▸), whereas the Raman tensors, required for the intensity calculations, were obtained via numerical differentiation of the forces in the application of small electric fields (Bastonero & Marzari, 2024 ▸). Computational details can be found in Section 2.8.

### 2.6. X-ray synchrotron total scattering

Total scattering data were collected using beamline P02.1 at PETRA-III, DESY, Hamburg (Dippel *et al.*, 2015 ▸) with a fixed energy of 60 keV [λ = 20.734(2) pm]. The beamline was equipped with a Varex XRD 4343CT detector (pixel size 150 × 150 µm, 2880 × 2880 pixels). Each sample was measured in 1 mm Kapton capillaries and exposed to radiation for 300 s within a setup particularly optimized for rapid *in-situ* measurement. PDF data processing was conducted using the *PDFGetX3* software (Juhás *et al.*, 2013 ▸). For all samples, *Q*_max_ was set to 1.95 nm^−1^. Structure model fitting against PDF data was performed using *PDFgui* (Farrow *et al.*, 2007 ▸). During the refinement process, instrumental parameters *Q*_damp_ and *Q*_broad_ were refined to the CeO_2_ standard dataset, and then kept fixed with *Q*_damp_ = 0.035693 and *Q*_broad_ = 0.001 for all the samples. The scale factor, lattice parameters, atomic displacement parameters (ADPs), atomic motion correlation factor and atomic coordinates were refined. The representative processed data *I*(*Q*), *S*(*Q*), *F*(*Q*) and *G*(*r*) of PFO are shown in Fig. S2. Stack plots of *I*(*Q*) and *S*(*Q*) for all the samples are given in Fig. S3.

### 2.7. DFT–PDF refinement

**Figure 2:**
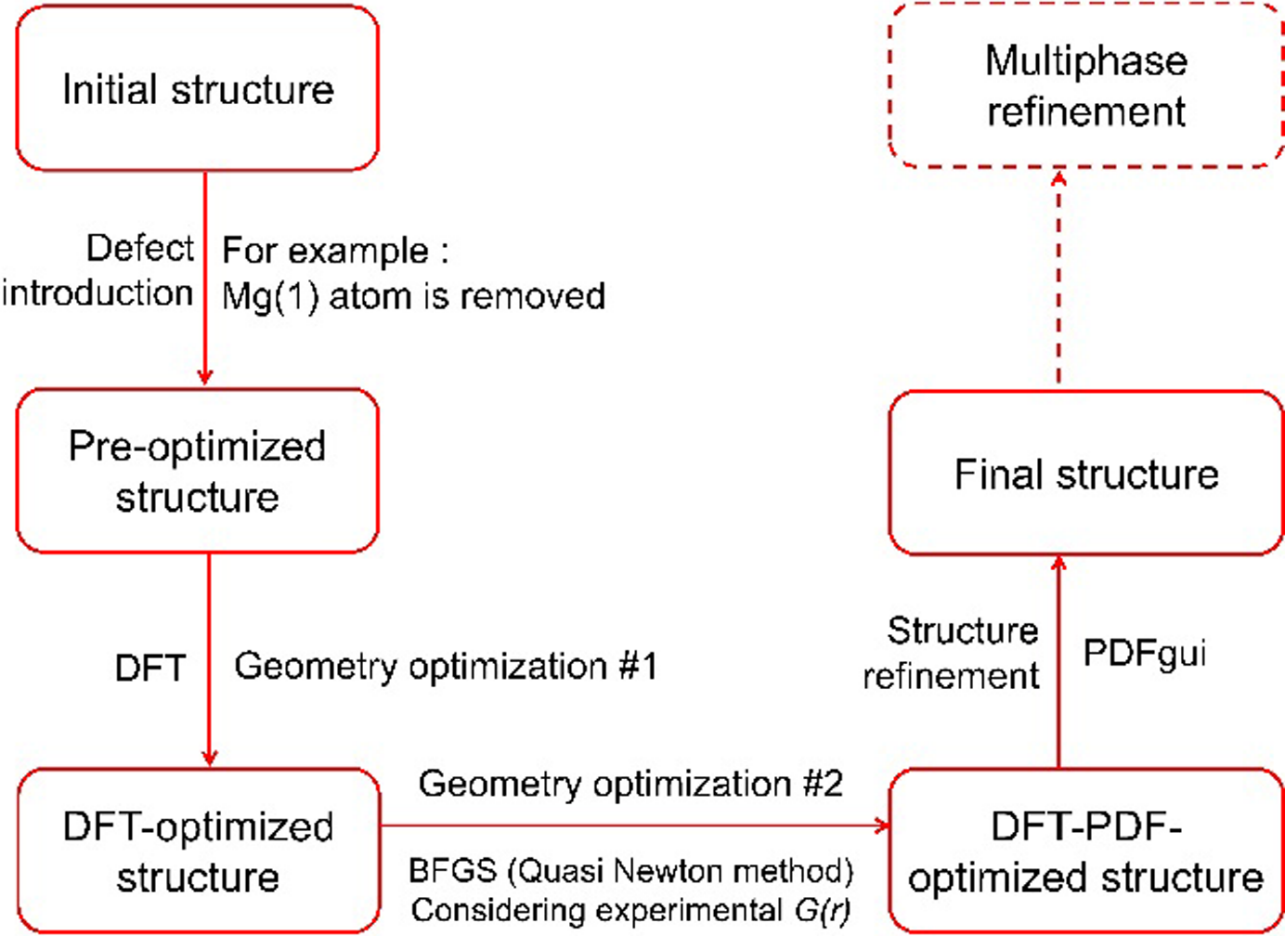
Schematic workflow maintained during the DFT–PDF refinement for different forsterite structures.

Combined DFT–PDF refinements of defective forsterite in the spirit of Dononelli (2023 ▸) and Kløve *et al.* (2023 ▸) were performed. Instead of globally optimizing the structure with the *GOFEE* algorithm (Bisbo & Hammer, 2020 ▸, 2022 ▸; Kløve *et al.*, 2023 ▸), several types of defects, namely vacancies and Frenkel and Schottky defects, were introduced to each atom site during the simulations. DFT was used as a tool to optimize every defect-type structure to their local minimum in the potential energy surface. The geometry-optimized structures were further optimized with a BFGS algorithm by considering the *G*(*r*) data from measurements and minimizing the *R*_PDF_. Finally, the structures were refined against the experimental data using *PDFgui*. The schematic workflow of DFT–PDF refinements is illustrated in Fig. 2 ▸.

Local structure optimizations have been performed using the electronic structure code *GPAW* (Enkovaara *et al.*, 2010 ▸) in the framework of the atomistic simulation environment (Larsen *et al.*, 2017 ▸). The exchange-correlation interaction was treated by the generalized gradient approximation (GGA) using the Perdew–Burke–Ernzerhof functional (Perdew *et al.*, 1996 ▸) with a 3 × 5 × 3 *k*-points sampling of Monkhorst & Pack (1976 ▸). Note that these calculations were not meant to provide very precise energetics or exact bond lengths. All structures optimized with such settings are later post-processed in PDF–Rietveld refinements to fit to experimentally observed bond lengths.

### 2.8. Energy calculations

To verify the favorite defective intrinsic candidate, the *ab initio* formation energy was investigated using DFT calculations. Exploiting the supercell approach, the defect formation energy in a charge state *q* can be computed as (Zhang & Northrup, 1991 ▸; Van de Walle *et al.*, 1993 ▸; Alkauskas *et al.*, 2011 ▸; Freysoldt *et al.*, 2014 ▸):
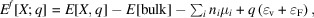
where *E*[*X, q*] is the total energy of the supercell calculation of defect *X* in the charge state *q* and *E*[bulk] is the total energy of the pristine crystal structure scaled to match the size of the defective supercell. Each defect is referenced to a chemical potential *μ_i_* corresponding to its species *i*, while the integer *n_i_* indicates the atoms of type *i* in excess (*n_i_* > 0) or removed (*n_i_* < 0). For charged states, the chemical potential for the extra electrons is given by the Fermi energy ɛ_F_ with respect to the valence band maximum ɛ_v_ of the pristine bulk supercell (Komsa *et al.*, 2012 ▸). The Fermi energy can be found by the condition of charge neutrality at a specific temperature when all the relevant defects are considered. In the following we consider ɛ_F_ = 0. To understand the defect formation in the diluted limit (very low defect concentrations), an additional correction term needs to be added due to the periodic boundary conditions, which is described in more detail in the supporting information.

Different defect types along with their nominal and neutral charge states, as well as both relaxed and non-relaxed geometries of the supercells, were thoroughly investigated. Four different supercell sizes of 2 × 1 × 2, 3 × 1 × 2, 3 × 2 × 2 and 3 × 3 × 2 were selected, as well as the single unit cell. For the interstitials, an algorithm introduced by Zimmermann *et al.* (2017 ▸) was used to find suitable atomic positions; 11 different positions were found for each species as possible candidates. Interestingly, the interstitials proposed by this pure geometric analysis for the magnesium atoms are found to be in tetrahedral coordination, as found in Walker *et al.* (2009 ▸), but here without the explicit energy calculation. The vacancies were instead generated using the space-group symmetries of forsterite, which greatly limits the number of positions.

To carry out the calculations, the *Quantum ESPRESSO* package (Giannozzi *et al.*, 2009 ▸, 2017 ▸, 2020 ▸) was used and the *PBEsol* (Terentjev *et al.*, 2018 ▸) functional was employed using pseudo-potentials from the precision *SSSP* library (version 1.1; Prandini *et al.*, 2018 ▸). The wavefunction and charge-density expansions were truncated with an energy cutoff of 80 and 960 Ry, respectively. The Brillouin zone was sampled using a uniform Monkhorst–Pack grid with a 4 × 2 × 3 *k*-points mesh. The geometry and atomic positions of forsterite were therefore relaxed until the total energy and forces were below 10^−6^ Ry atom^−1^ and 10^−5^ Ry Bohr^−1^, respectively. Supercell calculations were carried out using a gamma-point sampling, after having verified that the total energy changed by only 2 meV atom^−1^ for a 2 × 1 × 2 supercell. The geometry and the atomic positions of each defective supercell were optimized with lower thresholds for the total energy and forces of 10^−4^ Ry atom^−1^ and 10^−3^ Ry Bohr^−1^, respectively.

## 3. Results and discussion

### 3.1. Synthesis

Impure forsterite was obtained from BM synthesis with 7 (IFO-7) and 12.5 Hz (IFO-12). On the other hand, PFO was successfully obtained by RSC synthesis (Zampiva *et al.*, 2017 ▸) (RFO) and BM synthesis at 15 Hz (PFO). Both synthesis techniques yielded white forsterite powder. To introduce defects into the material, the attained PFO powder was mechanically post-processed by BM at 15 Hz for 1 h resulting in CFO. CFO possesses a slightly grayish color, either due to trace amounts of WC abraded from the mill, or suggesting the presence of defects. However, we estimated the amount to be lower than the detection limit [0.5(1) wt%] as we cannot observe any WC signal in the XRD (nor Raman) data. Finally, a small amount of CFO was re-calcined at 1373 K and a white powder of HFO with the expected lower defect concentrations was obtained. The synthesized samples and their respective IDs are listed in Table 1 ▸. Detailed information about phase quantification for impure forsterites determined from Rietveld refinements is provided in the Section 3.2.

### 3.2. X-ray powder diffraction

**Figure 3:**
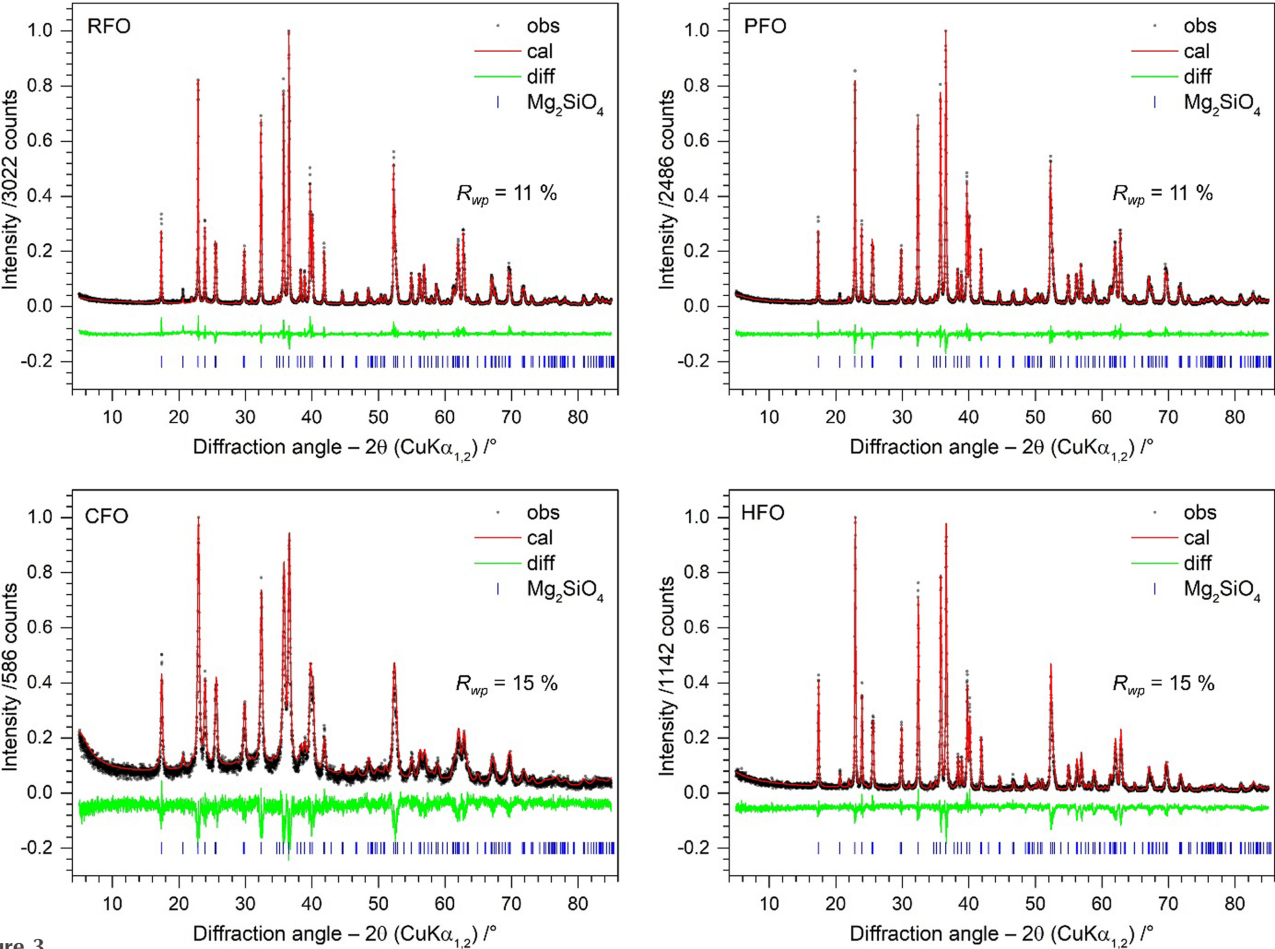
XRPD data Rietveld plots of different forsterites.

XRPD data Bragg–Rietveld refinements confirm that IFO-7 contains impurities of MgO, MgSiO_3_ and SiO_2_ in two modifications (α-cristobalite and α-quartz), whereas IFO-12 possesses only MgO as a minor impurity. This indicates that the BM frequencies of 7 and 12.5 Hz are not sufficient to form an intimate mixture of the reactants before the calcination process. On the contrary, pure forsterite was obtained from RSC (RFO) and BM (PFO) synthesis at 15 Hz. All reflections in the diffraction pattern of both samples can be indexed to olivine-type Mg_2_SiO_4_ with the space group *Pbnm* (Müller-Sommer *et al.*, 1997 ▸). The mechanically treated sample (CFO) is also characterized as a pure forsterite. However, broadening of the Bragg reflections along with significantly lower intensity maxima is observed, as shown in Fig. 3 ▸. Moreover, CFO exhibits notably lower ACS [25(1) nm] and DC [60(5) %] compared with those of PFO [ACS = 77(1) nm and DC = 98(5) %]. Inversely, the microstrain is increased from 0.031(1) to 0.140(4) upon BM. The re-calcination process of CFO led to re-crystallization, forming a crystalline forsterite with an ACS, ɛ_0_ and DC of 77(2) nm, 0.140(4) and 90(5) %, respectively, like those of PFO. Comparable values are observed when analyzing the ACS using the EnvACS (Gesing & Robben, 2024 ▸) approach. Nevertheless, the CSD provides additional information on the defect formation and the respective defect healing. For the synthesized samples (RFO and PFO) the CSD is narrow whereas a much broader CSD is observed for CFO. This is not surprising, as it is assumed that not only are defects introduced, but during the reduction of the ACS, not all crystallites are homogeneously destroyed due to crystallite cracking. For the heated CFO portion resulting in the HFO sample, it is obvious that the distribution narrows again by a factor of two but did not reach the narrow distribution of the as-synthesized PFO. Interestingly, meaningful results could only be obtained by also refining a scale factor in the EnvACS (Gesing & Robben, 2024 ▸) approach, which would represent the distribution of two different phases, namely the crystalline forsterite and the amorphous forsterite, respectively. The scale factors obtained correlate quite well with the DC obtained by the Bragg–Rietveld refinements. A complete list of XRPD characterization results is given in Table 2 ▸. The stack plots of XRPD patterns of all samples can be seen in Fig. S4.

Refined forsterite crystal data, along with comparative literature (Smyth & Hazen, 1973 ▸), are presented in Table S1 of the supporting information. The respective Bragg–Rietveld refinements converged with lower *R*_wp_ values for RFO (11 %) and PFO (11 %) compared with CFO (15 %) and HFO (15 %) (see Fig. 3 ▸ and Table S1). Moreover, structure refinements indicate that RFO and PFO can be classified as defect-poor forsterites, as their refined atomic positions possess only small changes (Δ*z* ≤ 0.003) compared with pristine forsterite. In contrast, noticeable structural changes are observed in both HFO and CFO. As an example, the O(3) atom in HFO slightly deviates from its initial position (Δ*z* ≤ 0.010) while CFO shows even stronger changes (Δ*z* ≤ 0.018). The strength of these observed structural changes is proportional to the expected defect concentration in the crystal, which is described in more detail in Section 3.3.

### 3.3. Raman spectroscopy

**Figure 4:**
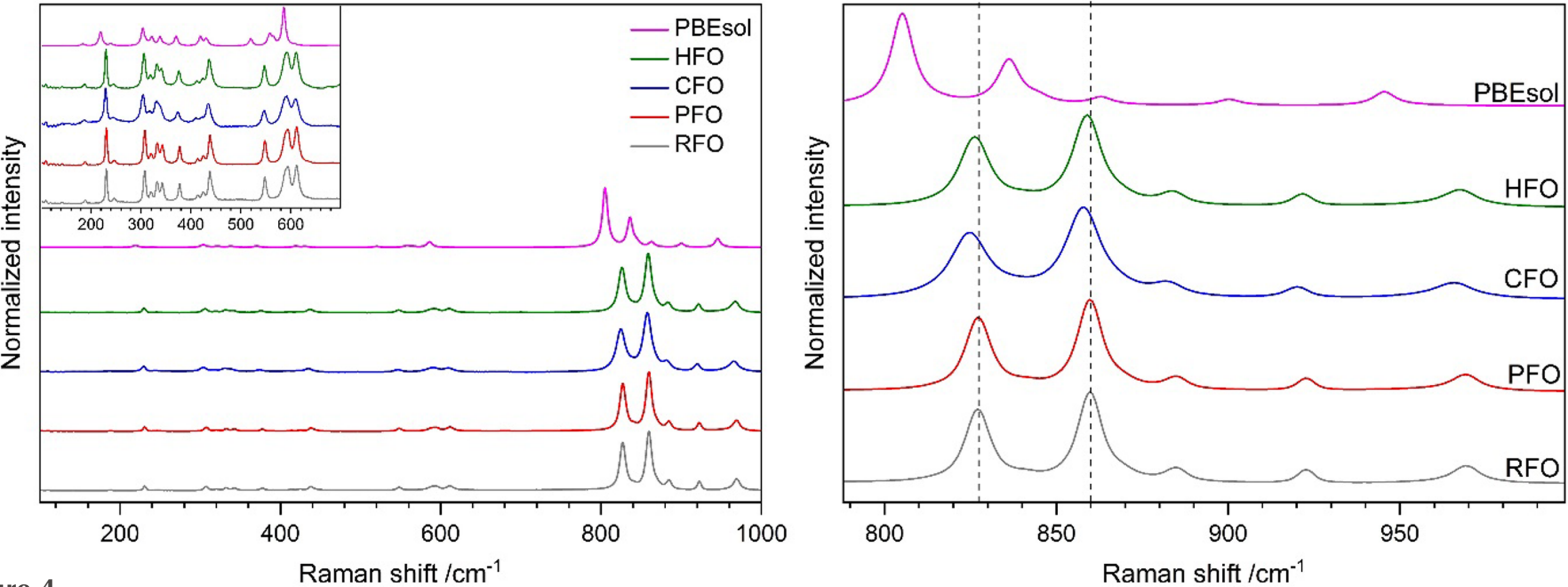
Raman spectra of different forsterites collected under ambient conditions and the *PBEsol* calculation (left). Magnified view of the high-frequency region (right); vertical dashed lines are a guide for the eye.

The factor group analysis predicts that orthorhombic Mg_2_SiO_4_ has 84 normal vibrational modes (11 A_g_ + 11 B_1g_ + 7 B_2g_ + 7 B_3g_ + 10 A_u_ + 10 B_1u_ + 14 B_2u_ + 14 B_3u_), among which A_g_, B_1g_, B_2g_ and B_3g_ modes are Raman active (Iishi, 1978 ▸; Hofmeister, 1987 ▸). Raman spectra of different forsterites are shown in Fig. 4 ▸. Peak fitting was performed for each experimental spectrum, representatively shown in Fig. S5 for PFO. The peak maxima along with comparative experimental reference data (Kolesov & Geiger, 2004 ▸) and theoretical calculations (Stangarone *et al.*, 2017 ▸; McKeown *et al.*, 2010 ▸) are given in Table S2. The observed band frequencies are in good agreement with those of the reported ones (Kolesov & Geiger, 2004 ▸; Stangarone *et al.*, 2017 ▸; McKeown *et al.*, 2010 ▸) and our own theoretical calculation. Typically, Raman spectra of olivine-type Mg_2_SiO_4_ can be classified into three regions: <400 cm^−1^, 400–700 cm^−1^ and >700 cm^−1^. The lower region bands are attributed to the vibrational modes from Mg [*M*(2) site] and negligible contribution from lighter silicon (Stangarone *et al.*, 2017 ▸; Chopelas, 1991 ▸). Peaks between 400 and 700 cm^−1^ are mainly contributed from bending motion of the Mg(2)—O bonds (Stangarone *et al.*, 2017 ▸). The high-frequency region (>700 cm^−1^) can be attributed to the internal Si—O stretching vibrations of the SiO_4_ tetrahedra (Chopelas, 1991 ▸; Stangarone *et al.*, 2017 ▸). The most dominant characteristic of the forsterite spectral range lies at around 820 and 860 cm^−1^ (Chopelas, 1991 ▸; Iishi, 1978 ▸; Wang *et al.*, 1995 ▸, 2004 ▸).

Some vibrational features from optical phonons are clearly distinguishable between the defect-poor samples (RFO and PFO) and the defect-containing samples (CFO and HFO). Global red shifts of ±1 cm^−1^ along with peak-broadening (ΔFWHM ≤ 2 cm^−1^) are observed in HFO. Moreover, greater red shifts of approximately 3(1) cm^−1^ as well as peak broadening (ΔFWHM ≤ 4 cm^−1^) are observed in CFO. The dominant two intense modes related to Si—O are further shifted to 825(1) and 857(1) cm^−1^, with FWHMs of 11(1) and 13(1) cm^−1^, respectively. In general, peak shifting and broadening in Raman spectra can be attributed to crystallite size effects and the degree of disorder in a structure (Swamy *et al.*, 2006 ▸; Islam *et al.*, 2005 ▸; Gouadec & Colomban, 2007 ▸; Demtröder, 2008 ▸). Here, the Raman peak broadening and shifts are proportional to the defect concentration in the structure. This finding further indicates that the CFO sample exhibits local structural disorder with the highest concentration.

### 3.4. PDF analysis

**Figure 5:**
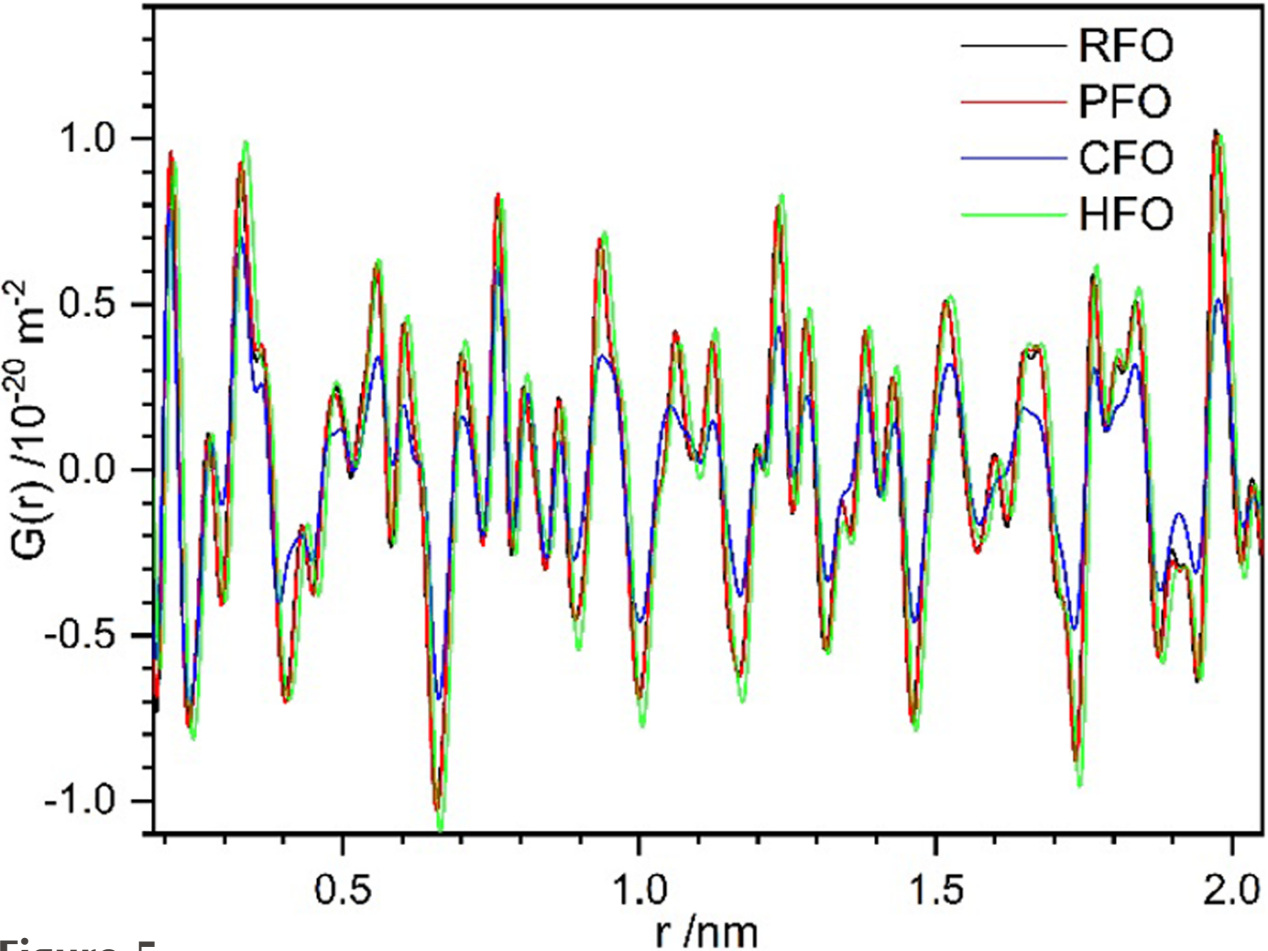
Observed *G*(*r*) for different forsterites.

To further investigate the defects and local structures of the samples, total scattering experiments were carried out (beamline P02.1 at PETRA-III, DESY, Hamburg). The analysis of the total scattering data allows the extraction of information from both Bragg and diffuse scattering contributions. The Bragg scattering contribution can be analyzed by the conventional approach in reciprocal space and provides information on the average and long-range periodic structure, whereas the diffuse scattering which lies between and beneath the Bragg reflections (Egami & Billinge, 2003 ▸) yields information regarding the short-range order and local structure deviations. Each measurement was integrated, background corrected and Fourier transformed to obtain the reduced PDF *G*(*r*). The *G*(*r*) describes the probability of finding two atoms separated by a distance of *r* (Teck *et al.*, 2017 ▸). Although the observed PDFs [*G*(*r*)] of RFO, PFO and HFO are very similar, that of CFO shows significant discrepancies (*e.g.* broadened signals, clear shoulders and lower intensities) as shown in Fig. 5 ▸.

**Figure 6:**
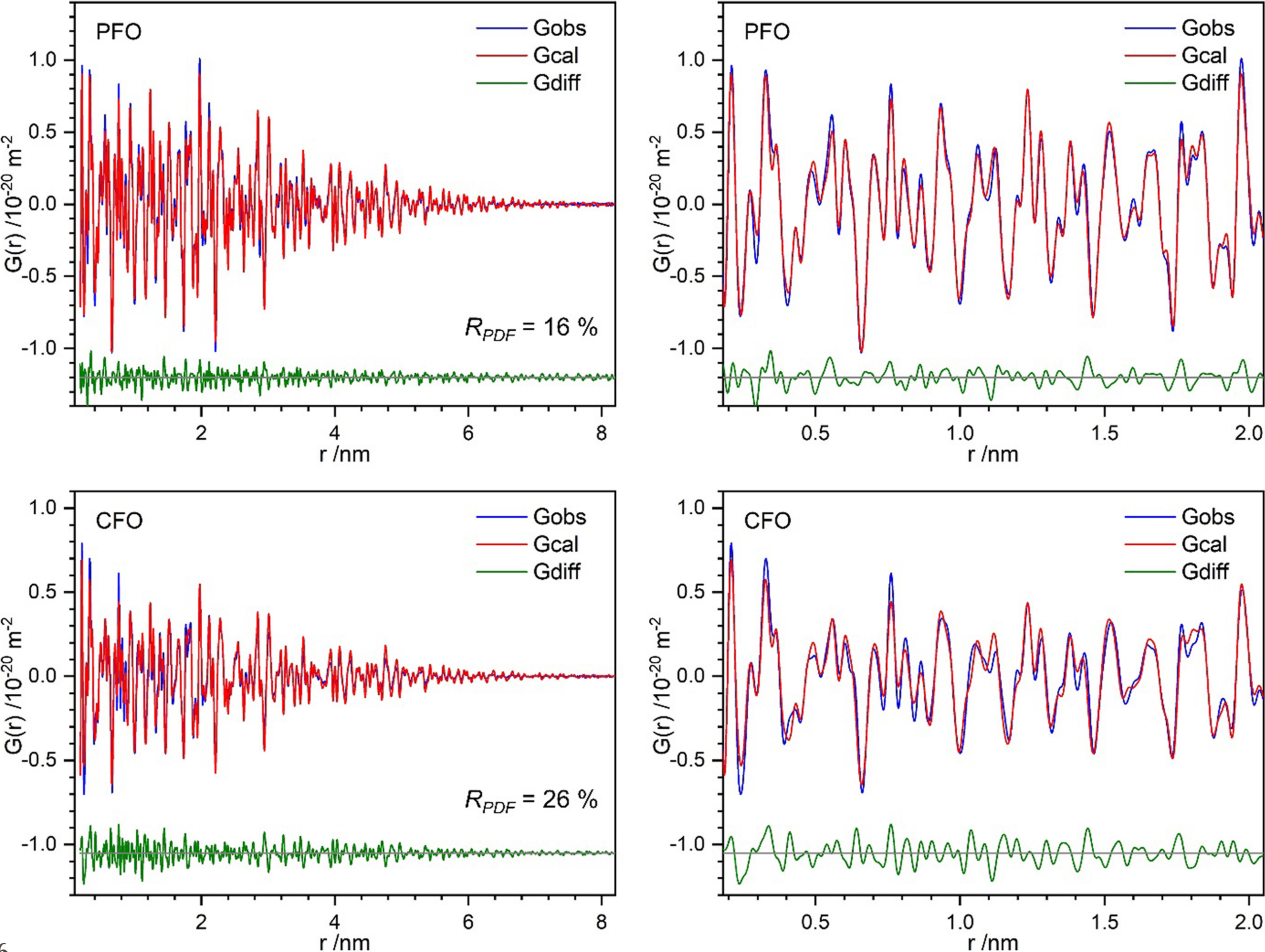
Representative PDF–Rietveld refinement plots of PFO and CFO in the long (left) and short to medium (right) range.

Defect-free, symmetry-constrained structural models of Mg_2_SiO_4_ were fitted against the experimental PDFs using a small-box modeling approach including symmetry constraints (PDF–Rietveld). Representative PDF–Rietveld refinements of the investigated forsterites are shown in Fig. 6 ▸. The refinements of CFO converged with *R*_PDF_ = 26 %, significantly higher than those of RFO, PFO (*R*_PDF_ = 16 %) and HFO (*R*_PDF_ = 18 %). The higher *R*_PDF_ of CFO indicates that a simple PDF–Rietveld refinement using an ideal average crystal structure model struggles to describe the defect-rich local nature of the post-milled sample. As such, a more advanced defect-rich structure model based on DFT–PDF refinements is proposed in this work, described in more detail in Section 3.5.

The bond lengths obtained from Bragg–Rietveld and PDF–Rietveld refinements are compared in Table S3 within each of the forsterite samples. Based on Bragg–Rietveld refinements, the average bond lengths [further noted as 〈Mg(1)—O〉, 〈Mg(2)—O〉 and 〈Si—O〉] of RFO and PFO are virtually identical, whereas the bond lengths of HFO only differ by a maximum of 0.1 pm. Interestingly, 〈Mg(1)—O〉 and 〈Mg(2)—O〉 of CFO are slightly longer than those of the defect-poor forsterites [Δ1 pm for 〈Mg(1)—O〉 and Δ0.5 pm for 〈Mg(2)—O〉]. On the contrary, the 〈Si—O〉 of CFO is the shortest among all forsterites (Δ2 pm). As a consequence, the bond valence sum (BVS) (Brese & O’Keeffe, 1991 ▸) of Si in the CFO is found to show over bonding [4.19(2) v.u.].

Unlike the Bragg–Rietveld refinements, which suggest shorter 〈Si—O〉 bond lengths for CFO, the 〈Si—O〉 bonds determined through PDF–Rietveld refinements consistently display similar values. This results in an Si BVS of 3.84(3) v.u. across all forsterites. We attribute the different interatomic distances obtained from PDF–Rietveld and Bragg–Rietveld analyses to ACS limitation effects of the short synchrotron wavelength due to maximal observable average crystallite size [MOACS (Gesing & Robben, 2024 ▸)] and the repeated observation. Furthermore, Rietveld refinements often describe local distortions, such as atom displacement, with an increase in the Debye–Waller factor (Abeykoon *et al.*, 2009 ▸).

### 3.5. DFT–PDF refinement

#### 3.5.1. Single-phase refinement

Small box (single unit cell): in Section 3.4 the fitting results against crystalline Mg_2_SiO_4_ for all samples have been given. To receive a better fit of the structure model against the experimental PDF data of CFO, DFT-assisted PDF refinements (DFT–PDF) were implemented. As an initial step, a geometry-optimized structure model of Mg_2_SiO_4_ without defects (GOSWD) was selected. Single-phase DFT–PDF refinements using this symmetry-free structure model showed a slightly better fit (*R*_PDF_ = 23 %) compared with the original (symmetry-constraint) model for defect-free, crystalline Mg_2_SiO_4_ (*R*_PDF_ = 26 %). Then, 17 structure models containing vacancy, Schottky and Frenkel defects (see Fig. S1 of the supporting information) were generated from the DFT–PDF workflow (Section 2.7). Furthermore, each DFT–PDF-generated structure model was individually selected for DFT–PDF refinement. Overall, the new defective structure models gave an *R*_PDF_ in the range 22–28 %, where defects involving oxygen show the lowest values.

Large box (2 × 2 × 2 unit cells): to realize lower concentrations of defects, larger systems (2 × 2 × 2 unit cells) were employed. To begin with, DFT–PDF refinements of a pristine 2 × 2 × 2 structure without further structure variation gave *R*_PDF_ = 37 %, while its GOSWD converged with *R*_PDF_ = 27 %. The significant mismatch observed between these refinements offers additional indications that the crystalline structure is unable to accommodate the defect-rich characteristics of the CFO sample. As before, 17 defective 2 × 2 × 2 models containing vacancy, Frenkel and Schottky defects were generated from DFT–PDF optimization. The refinements converged with an *R*_PDF_ in the range 19–22 %. Nine of them (four different vacancies, two Frenkel and three Schottky defects) have almost identical *R*_PDF_ values of 19 %.

Point defect (2 × 1 × 2 unit cells): in a third approach, charge defect analysis was in focus. Both charged and neutral defects are considered for this calculation, for which the size of 2 × 1 × 2 unit cells was selected. In total, ten defective 2 × 1 × 2 structure models were investigated: Mg interstitial and vacancy, O interstitial and vacancy, as well as Si interstitial. The refinements converged with *R*_PDF_ = 25–28 %, showing trends like the values for the single unit cells used. Table S4 summarizes the *R*_PDF_ values of all defective structure candidates.

#### 3.5.2. Stability of defective structure candidates

The calculated formation energies of the defective structure candidates are reported in Table S4. Positive numbers indicate metastable or unfavorable structures, whereas negative values indicate spontaneous or favorable formation of the structures. It can be observed that most of the structures have positive formation energies. Schottky defects in particular show the highest formation energies (>8 eV) and therefore those structures should be discarded as possible candidates. Table 3 ▸ shows selected defective structure candidates (CIFs can be found in the supporting information) with formation energies <5 eV. Note, the formation energies in Table 3 ▸ were not corrected to account for the formation of a single defect in the dilute limit, since we expect high defect concentrations in CFO. A complete list of formation energies at different defect concentrations, to extrapolate the dilute limit, can be found in Table S5.

The interpretation of formation energies is not straightforward, since they strongly depend on the calculation scheme and the defect concentration/size of the simulation cell. Earlier studies by Walker *et al.* (2009 ▸, 2003 ▸) did not consider the possible interaction between the defects (*i.e.* they performed a ‘mere’ energy difference, hence the interaction of charged defects is long-ranged and sizeable). As an improvement, we extrapolated the free energy of formation to the infinite volume, *i.e.* to the dilute limit (using the formula shown in the supporting information). Nevertheless, similarly we found that Mg(1) Frenkel defects are energetically stable with a formation energy of 2.98 eV. In addition, we performed vacancy and interstitial supercell calculations with charged point defects and found comparable results to the available literature (Walker *et al.*, 2009 ▸, 2003 ▸). At high defect concentrations, both Si^4+^ and Mg^2+^ interstitials seem to be the most energetically favorable. However, oxygen vacancies are found to be the most favorable defects in the extrapolated dilute limit (very low concentration). This may be due to the usage of a GGA functional, which tends to underestimate the bond strength of the oxygen molecule. Table S5 summarizes the formation energy of charge defect structures computed using different fixed supercell sizes and extrapolated dilute limits.

#### 3.5.3. Multi-phase refinement

**Figure 7:**
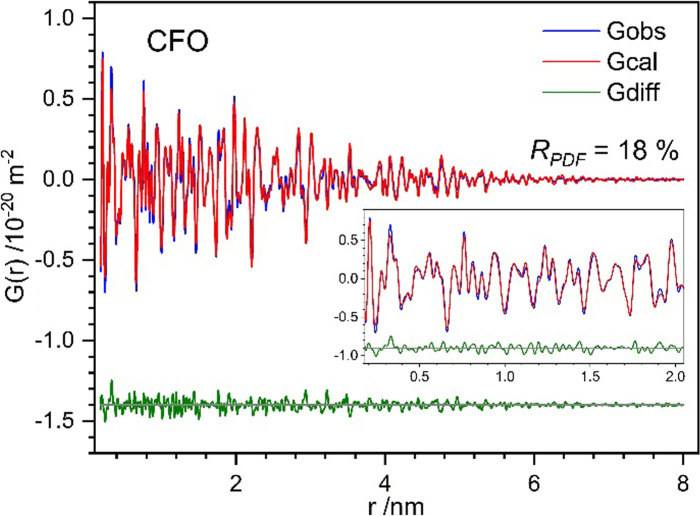
Multi-phase DFT–PDF refinement plot of CFO in the long and short to medium (inset) range.

**Figure 8:**
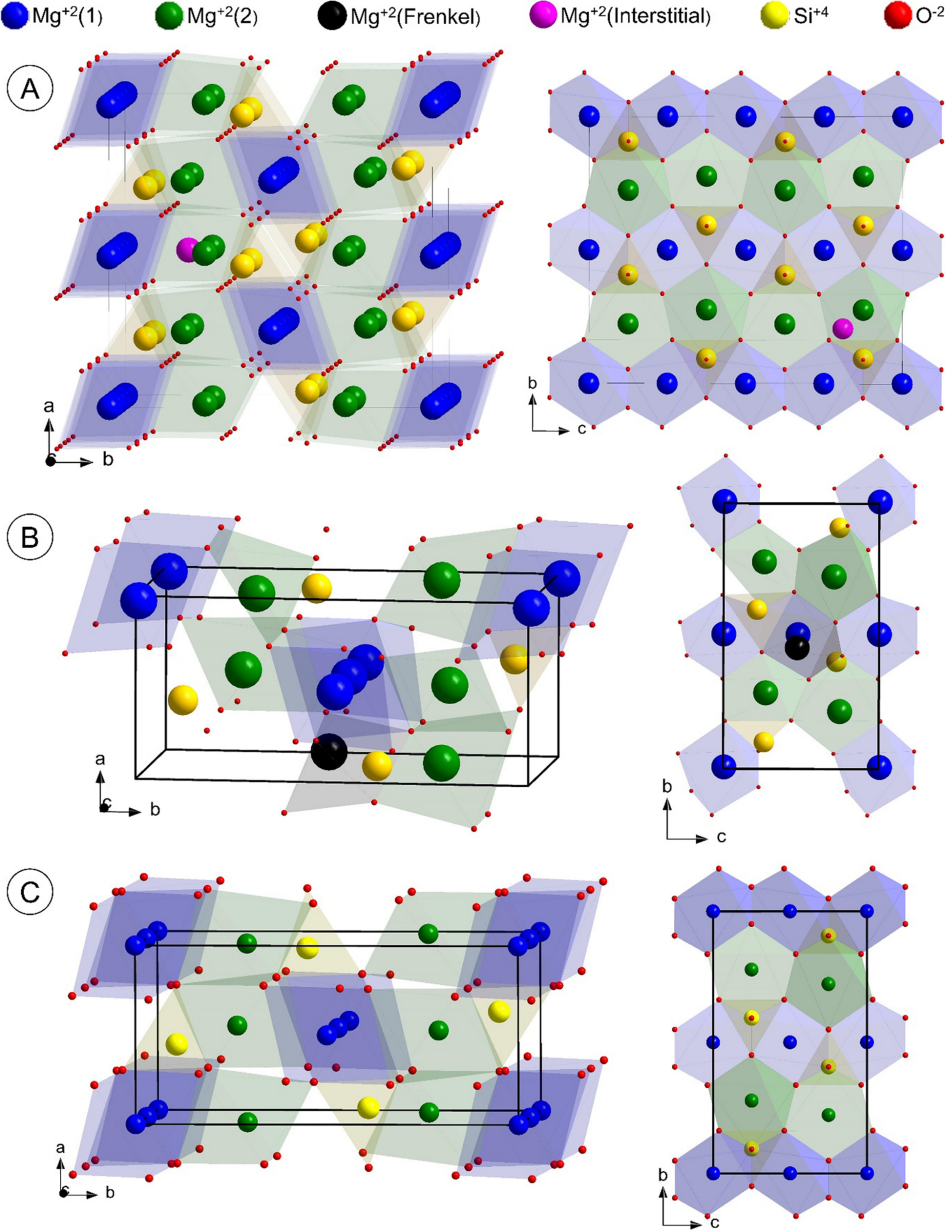
DFT–PDF optimized crystal structures of (*a*) 2 × 1 × 2 Mg^2+^ interstitial and (*b*) single unit cell Mg(1) Frenkel defects in comparison with the (*c*) pristine forsterite structure.

Ultimately, all structure motifs with favorable energy (as listed in Table 3 ▸) were used in combined multi-phase refinements. In principle, the respective defective structure is treated as a secondary phase alongside the crystalline one (GOSWD). The refined defective phases showing a negative scale factor were removed one at a time from the refinements. The best fit is finally achieved using a combination of a single unit cell of GOSWD and Mg(1) Frenkel, along with 2 × 1 × 2 Mg^2+^ interstitial defect structures which converge to *R*_PDF_ = 18 %, as illustrated in Fig. 7 ▸. This fitting indicates that CFO consists of 67(3) wt% GOSWD, 23(3) wt% Mg Frenkel and 10(3) wt% of Mg^2+^ interstitial. The optimized defective forsterite structure models are shown in Fig. 8 ▸. Note, however, that any defect summarized in Table 3 ▸ might be present in defective Mg_2_SiO_4_, but not in the CFO sample with defects mechanically induced by BM. Although Si^4+^ interstitials might be favorable from an energetic point of view, this motif does not improve the PDF fit of CFO. Therefore, the presence of this type of defect is rather unlikely, and is probably prevented by the atmospheric reaction conditions leading to a high concentration of oxygen-rich phases.

## 4. Conclusions and outlook

Structural differences between defect-poor and defect-rich forsterite (Mg_2_SiO_4_) were investigated. Mechanically induced defect-rich forsterite was obtained by BM of defect-poor (pristine) forsterite. Implementing PDF–Rietveld refinements on X-ray synchrotron data indicated a complex disorder structure in the defect-rich forsterite. Raman peak broadening and global red shifts complemented the structural features of the defective phases. The defect-rich structure models were simulated using the DFT–PDF method to better describe the disorder in the local structure. DFT–PDF refinements indicate that post-processed forsterite contains Mg Frenkel-type and Mg^2+^ interstitial defects with concentrations of 23(3) and 10(3) wt%, respectively. DFT calculations confirmed that the defective structure models are energetically stable. This finding is an important starting point to characterize and quantify defect-rich Martian regolith. Further investigations involving a larger number of phases are necessary as a stepwise strategy to structurally describe multi-phase Martian regolith. Additionally, a comparative study between radiation-induced defects and the mechanically induced defects described here would be of high demand to understand the mechanism of space weathering effects.

## Supplementary Material

Crystal structure: contains datablock(s) Mg2SiO4_CFO_USI_2121821137_Frenkel_Mg1, Mg2SiO4_CFO_USI_2121821137_GOSWD, Mg2SiO4_CFO_USI_2121821137_Interstitial_Mg_0, Mg2SiO4_CFO_USI_2121821137_Interstitial_Mg_+2, Mg2SiO4_CFO_USI_2121821137_Interstitial_O_0, Mg2SiO4_CFO_USI_2121821137_Interstitial_Si_+4, Mg2SiO4_CFO_USI_2121821137_Vacancy_O_+2, Mg2SiO4_CFO_USI_2121821137, Mg2SiO4_HFO_USI_2121821138, Mg2SiO4_PFO_USI_2121821136, Mg2SiO4_RFO_USI_2121821033. DOI: 10.1107/S2052252524009722/lt5071sup1.cif

Supporting figures and tables. DOI: 10.1107/S2052252524009722/lt5071sup2.pdf

Powder diffraction data. DOI: 10.1107/S2052252524009722/lt5071sup3.zip

CCDC references: 2391997, 2391998, 2391999, 2392000, 2392001, 2392002, 2392003, 2392004, 2392005, 2392006, 2392007

## Figures and Tables

**Table 1:** List of synthesized samples and their abbreviations

Sample ID	Material
RFO	RSC synthesis forsterite
IFO-7	Impure forsterite from 7 Hz BM
IFO-12	Impure forsterite from 12.5 Hz BM
PFO	Pristine forsterite from 15 Hz BM
CFO	Crushed forsterite (post-processed PFO)
HFO	Healed forsterite (re-calcined CFO)

**Table 2:** ACS and microstrain (ɛ_0_) of forsterites, and the DC of the synthesized samples obtained from Bragg–Rietveld refinements of XRPD data Additionally, the ACS and the CSD factor, correlating the smallest (0.01) and broadest (1) distribution of spherical crystallites, are given. Both values were refined with *a* fixed to 1 and a variable scale factor, considering perfect and reduced DC, respectively.

	Bragg–Rietveld					EnvACS		
Sample ID	Phase fraction /%		ACS /nm	ɛ_0_	DC /%	ACS /nm	CSD factor	Scale factor
IFO-7	Mg_2_SiO_4_	62(2)	58(1)	0.048(2)	95(5)	–	–	–
	MgO	23(2)	–	–	–	–	–	–
	MgSiO_3_	8(2)	–	–	–	–	–	–
	SiO_2_ α-cristobalite	6(2)	–	–	–	–	–	–
	SiO_2_ α-quartz	1(2)	–	–	–	–	–	–
IFO-12	Mg_2_SiO_4_	98(2)	61(1)	0.053(5)	93(5)	–	–	–
	MgO	2(2)	–	–	–	–	–	–
RFO	Mg_2_SiO_4_	100(2)	89(1)	0.027(1)	95(5)	85.5(5)	0.01(1)	1
						87.1(5)	0.01(1)	0.98(1)
PFO	Mg_2_SiO_4_	100(2)	77(1)	0.031(1)	98(5)	88.3(5)	0.01(1)	1
						89.0(5)	0.01(1)	0.99(1)
CFO	Mg_2_SiO_4_	100(2)	25(1)	0.140(4)	60(5)	33.3(2)	0.01(1)	1
						57.6(3)	0.17(1)	0.68(1)
HFO	Mg_2_SiO_4_	100(2)	77(2)	0.046(1)	90(5)	74.9(6)	0.01(1)	1
						75.4(4)	0.07(1)	0.95(1)

**Table 3:** Selected defective structure candidates along with their symmetry analysis upon cell relaxation, formation energy and *R*_PDF_ values GOSWD = geometry-optimized structure without defect, F = Frenkel, I = interstitial, V = vacancy. The structure model marked with * falls back to the pristine structure upon optimization.

Defective structure candidate	Unit-cell size	Symmetry analysis	Formation energy /eV	*R*_PDF_ /%
GOSWD	1	*Pbnm* (62)*	0.01	23
Mg(1) F	1	*P*1 (2)	2.98	25
Mg I +0	2 × 1 × 2	*P*1 (1)	3.73	27
Mg I +2	2 × 1 × 2	*P*1 (1)	−5.25	27
O I +0	2 × 1 × 2	*P*1 (1)	1.37	27
O V +2	2 × 1 × 2	*P*1 (1)	1.49	27
Si I +4	2 × 1 × 2	*P*1 (1)	−9.07	28
