# Supplementary material for: Synthesis, structural and spectroscopic characterization of defect-rich forsterite as a representative phase of Martian regolith
Source: IUCrJ. 2024 Oct 28;11(Pt 6):977–90. doi: 10.1107/S2052252524009722 (PMC11533994; doi:10.1107/S2052252524009722)
Supplement: Supplementary file 2 [file m-11-00977-sup2.pdf]

IUCrJ

Volume 12 (2025)

Supporting information for article:

Synthesis, structural and spectroscopic characterization of defect-rich forsterite as a representative phase of Martian regolith

Md. Izzuddin Jundullah Hanafi, Lorenzo Bastonero, M. Mangir Murshed, Lars Robben, Wilke Dononelli, Andrea Kirsch, Nicola Marzari and Thorsten M. Gesing

S1. Defect formation energy in the dilute limit

The dilute limit for (point) defects assumes that a single defect is present in an infinite perfect crystal. This is a good approximation when its concentration is limited to a few percentage (Freysoldt *et al.*, 2014). When the defect is charge, the simulation in periodic boundary conditions (PBC) comes with a number of cautions to take into account. The major issue is the slow decay of the long-range nature of the Coulomb interaction, which decays as the inverse of the cell size L (Komsa *et al.*, 2012), or equivalently as the inverse cube root of the cell volume Ω. To extrapolate the value in the dilute limit, one could adopt a correction scheme (Komsa *et al.*, 2012) or alternatively extract the value from a series of supercells calculations. The formation energy can be written as a function of the defective supercell volume (subject to PBC), as:

$$E^f[\Omega] = E^f{}_\infty + \frac{a}{\Omega^{1/3}} + \frac{b}{\Omega}$$

where $E^f{}_\infty$, a, and b are parameters to fit. In particular, $E^f{}_\infty$ represents the formation energy in the dilute limit, i.e. when $\Omega \rightarrow \infty$. Using the formation energies in **Table S5**, $E^f{}_\infty$ is extrapolated for all the presented charged defects. A rigorous treatment of the electrostatic should also account for the misalignment of the average potential with the reference pristine bulk (Freysoldt *et al.*, 2014). Nevertheless, this results in small energy corrections, usually lower than 1 eV/atom (Freysoldt *et al.*, 2014). Since our calculated formation energies are well separated in energy and considering we do not need such accuracy for screening the type of defects for the further refinement, we neglect this extra term in the prediction of the formation energy in the dilute limit in this study.

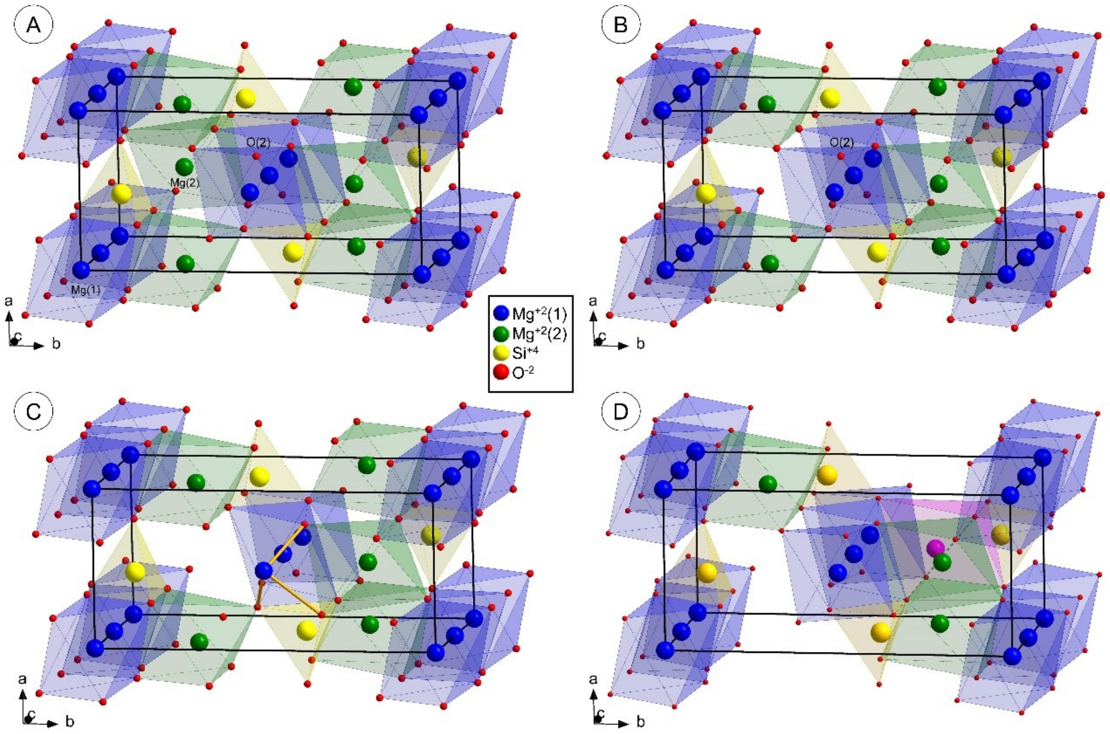

Figure S1 Crystal structure of **(A)** pristine forsterite, defective forsterite with **(B)** vacancy in Mg(2)-site. **(C)** Schottky Mg(2)-O(2) where both atoms are missing, and **(D)** Frenkel where Mg(2) is dislocated (coloured in pink) close to another Mg(2)-site.

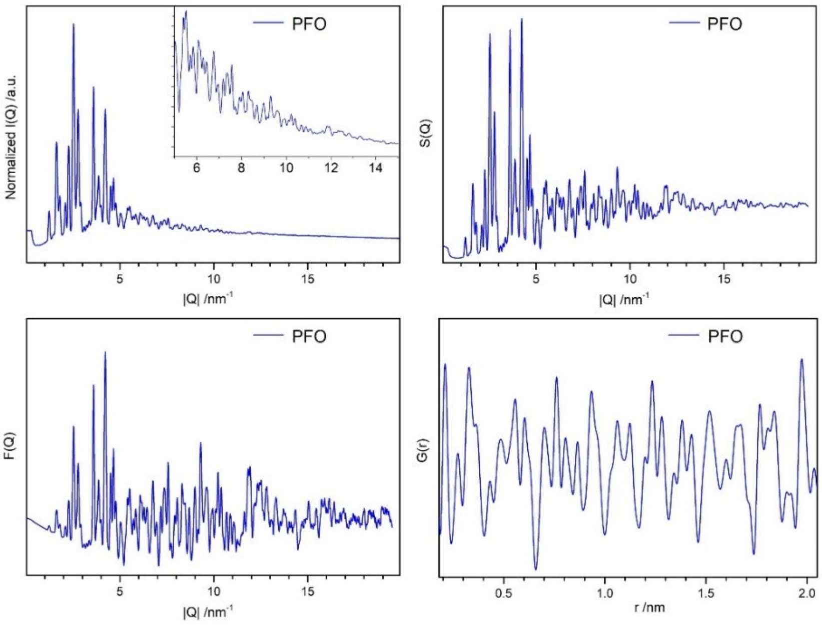

Figure S2 Powder X-ray diffraction data of PFO, showing plots of *I(Q)*, *S(Q)*, *F(Q)*, and *G(r)*.

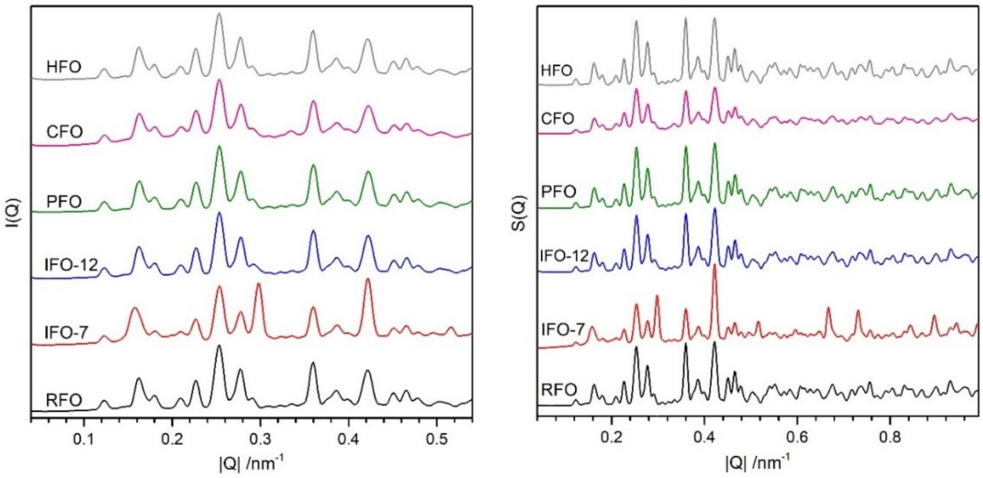

Figure S3 *I(Q)* and *S(Q)* of all synthesized samples.

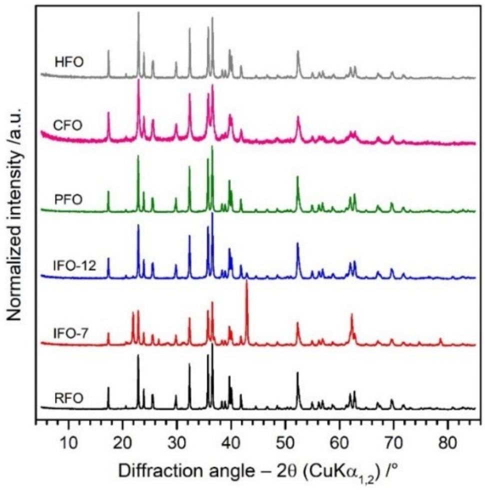

Figure S4 X-ray powder diffraction patterns of synthesized samples.

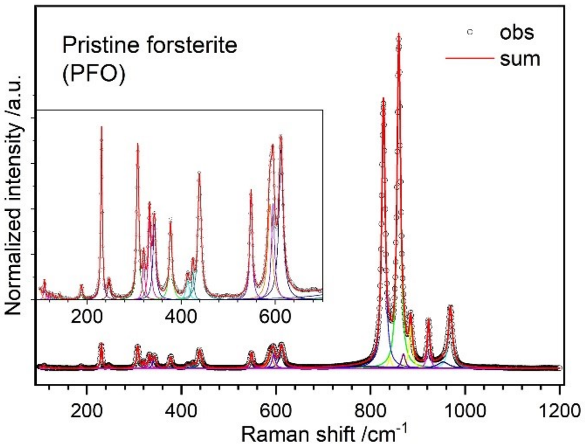

Figure S5 Representative peak fitting of a forsterite Raman spectrum synthesized by ball mill method.

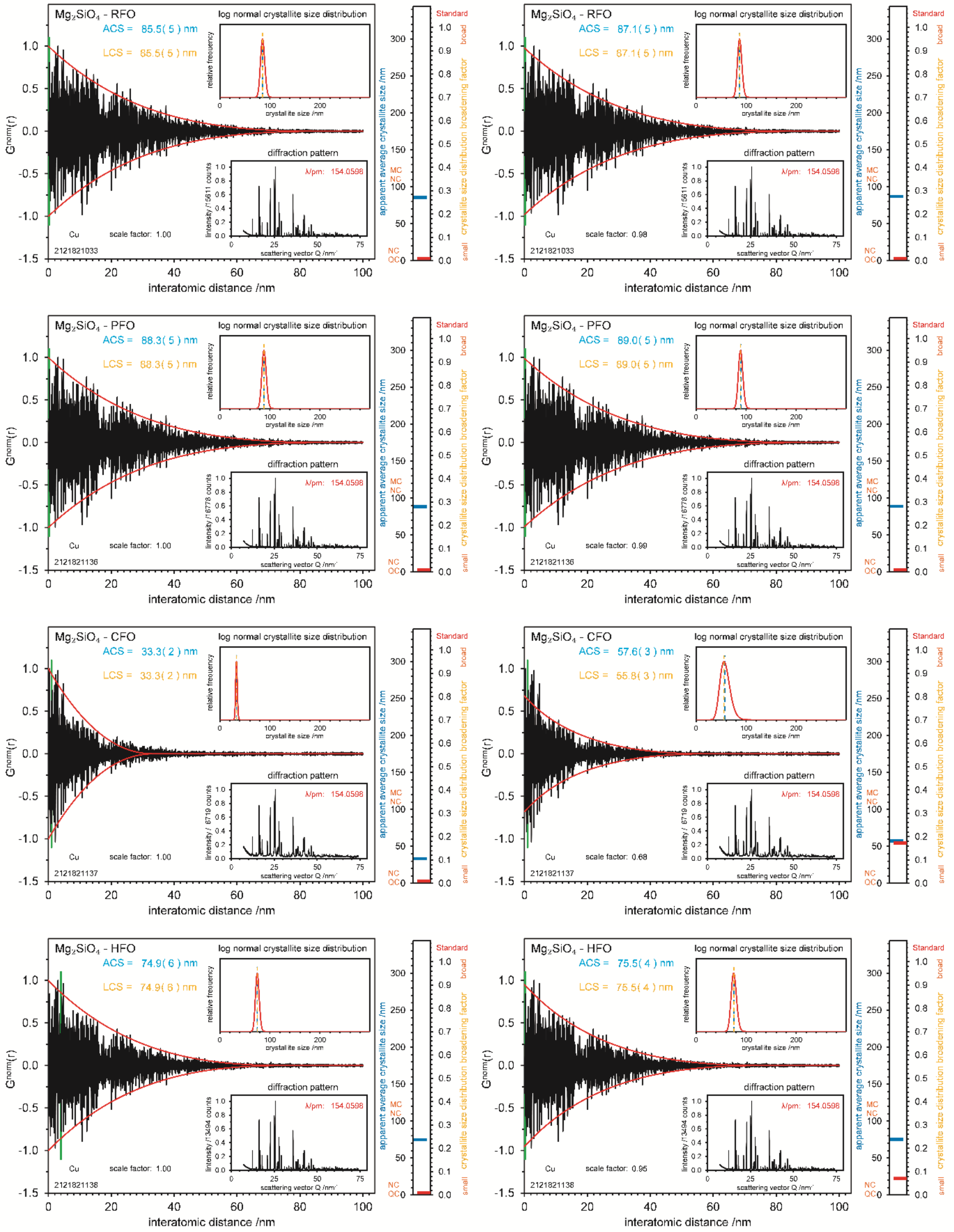

Figure S6 EnvACS plots of refinements without (left) and with (right) including a variable scale factor for RFO, PFO, CFO and HFO.

Table S1 Comparative metric and structural parameters of forsterites obtained from Bragg-Rietveld refinements of XRPD data.

Atom	Site	x	Y	Z	B_{iso} /10^4 pm^2
Literature (Smyth & Hazen, 1973), *Pbnm*, *a* = 475.6(1) pm, *b* = 1020.7(1) pm, *c* = 598.0(1) pm					
Mg(1)	4a	0	0	0	0.26(1)
Mg(2)	4c	0.9915(2)	0.2774(1)	0.25	0.22(1)
Si	4c	0.4262(1)	0.0940(1)	0.25	0.08(1)
O(1)	4c	0.7657(3)	0.0913(2)	0.25	0.27(2)
O(2)	4c	0.2215(4)	0.4474(2)	0.25	0.24(2)
O(3)	8d	0.2777(2)	0.1628(1)	0.0331(2)	0.27(2)
RFO sample, *Pbnm*, *a* = 475.15(1) pm, *b* = 1021.26(2) pm, *c* = 598.54(1) pm, R_{wp} = 11 %					
Mg(1)	4a	0	0	0	0.81(4)
Mg(2)	4c	0.9919(4)	0.2775(1)	0.25	0.81(4)
Si	4c	0.4272(3)	0.0941(2)	0.25	0.57(4)
O(1)	4c	0.7626(5)	0.0922(3)	0.25	0.40(5)
O(2)	4c	0.2195(5)	0.4500(3)	0.25	0.40(5)
O(3)	8d	0.2804(4)	0.1617(2)	0.0357(3)	0.40(5)
PFO sample, *Pbnm*, *a* = 475.30(1) pm, *b* = 1020.84(2) pm, *c* = 598.48(1) pm, R_{wp} = 11 %					
Mg(1)	4a	0	0	0	0.95(4)
Mg(2)	4c	0.9929(4)	0.2775(2)	0.25	0.95(4)
Si	4c	0.4268(3)	0.0940(2)	0.25	0.76(4)
O(1)	4c	0.76226)	0.0923(3)	0.25	0.54(5)
O(2)	4c	0.2205(6)	0.4501(4)	0.25	0.54(5)
O(3)	8d	0.2802(4)	0.1621(2)	0.0358(4)	0.54(5)
CFO sample, *Pbnm*, *a* = 475.46(5) pm, *b* = 1020.48(1) pm, *c* = 598.40(7) pm, R_{wp} = 15 %					
Mg(1)	4a	0	0	0	2.74(11)
Mg(2)	4c	0.9840(1)	0.2766(4)	0.25	2.74(11)
Si	4c	0.4300(9)	0.0983(4)	0.25	2.89(13)
O(1)	4c	0.7578(15)	0.0878(9)	0.25	2.20(14)

O(2)	4c	0.2211(15)	0.4490(11)	0.25	2.20(14)
O(3)	8d	0.2762(11)	0.1546(6)	0.0510(10)	2.20(14)
HFO sample, *Pbnm*, a = 475.24(2) pm, b = 1020.30(4) pm, c = 598.23(2) pm, R_{wp} = 15 %					
Mg(1)	4a	0	0	0	3.18(7)
Mg(2)	4c	0.9915(7)	0.2769(2)	0.25	3.18(7)
Si	4c	0.4275(5)	0.0949(3)	0.25	2.95(8)
O(1)	4c	0.7616(9)	0.0907(5)	0.25	2.68(9)
O(2)	4c	0.2162(8)	0.4518(6)	0.25	2.68(9)
O(3)	8d	0.2833(6)	0.1583(4)	0.0435(6)	2.68(9)

Table S2 Raman frequencies (cm^{-1}) of the deconvoluted peak maxima of different forsterites are compared with those of theoretical (PBEsol) and literature values.

Raman shift /cm^{-1}								
**RFO**	**PFO**	**CFO**	**HFO**	**(Kolesov & Geiger, 2004)**	**(Stangarone *et al.*, 2017)**	**(McKeown *et al.*, 2010)**	**PBEsol**	**Assignment**
-	-	-	-	-	-	-	171	B_{2g}
-	-	-	-	-	-	-	183	A_g
188(1)	188(1)	187(1)	187(1)	184	191	190	186	B_{3g}
-	-	-	-	-	-	-	216	B_{1g}
-	-	-	-	-	-	-	219	A_g
231(1)	231(1)	229(1)	230(1)	227	228	224	240	B_{2g}
247(1)	247(1)	241(1)	246(1)	243	234	246	250	B_{1g}
-	-	-	-	-	-	-	279	B_{3g}
307(1)	308(1)	303(1)	306(1)	304	304	-	304	A_g
-	-	-	-	-	-	-	310	B_{3g}
-	-	-	-	-	-	-	313	B_{1g}
320(1)	320(1)	319(1)	319(1)	316	315	317	318	B_{2g}
332(1)	333(1)	330(1)	332(1)	329	324	332	322	A_g
343(1)	343(1)	338(1)	341(1)	-	336	-	338	A_g
-	-	-	-	-	351	354	348	B_{1g}
-	-	-	-	-	-	-	360	B_{2g}
-	-	-	-	373	372	383	370	B_{3g}
377(1)	377(1)	374(1)	376(1)	-	378	-	375	B_{1g}
-	-	-	-	-	-	-	404	B_{3g}
414(1)	414(1)	412(1)	412(1)	421	424	-	419	A_g
424(1)	425(1)	422(1)	424(1)	434	432	433	430	B_{1g}

439(1)	439(1)	435(1)	437(1)	-	448	443	433	B_{2g}
-	-	-	-	-	-	-	520	A_g
548(1)	549(1)	544(1)	547(1)	544	556	534	556	B_{1g}
-	-	-	-	-	-	-	559	B_{2g}
589(1)	588(1)	-	-	588	594	574	566	B_{3g}
595(1)	595(1)	590(1)	591(1)	-	603	582	586	A_g
612(1)	612(1)	610(1)	611(1)	608	605	600	608	B_{1g}
-	-	-	-	-	-	-	805	A_g
827(1)	827(1)	825(1)	826(1)	824	819	821	817	B_{1g}
841(1)	841(1)	-	840(1)	838	834	-	836	A_g
860(1)	860(1)	857(1)	859(1)	856	857	852	845	B_{1g}
869(1)	869(1)	-	-	866	867	861	863	B_{2g}
885(1)	885(1)	882(1)	884(1)	882	886	880	900	B_{3g}
923(1)	922(1)	920(1)	922(1)	920	929	917	945	A_g
969(1)	969(1)	965(1)	967(1)	966	974	967	956	B_{1g}

Table S3 Selective interatomic distances (/pm) and their average values (<M-O>) in Mg_2SiO_4 obtained from Bragg- and PDF-Rietveld refinements along with the bond valence sum (BVS).

**Bond length**	**Method**	**Sample**			
		**RFO**	**PFO**	**CFO**	**HFO**
Si-O(1)	Bragg	162.0(1)	162.0(1)	159.2(1)	162.1(1)
	PDF	*162.0(2)*	*162.4(2)*	*161.4(3)*	*162.3(2)*
Si-O(2)	Bragg	163.8(1)	163.8(1)	160.8(1)	163.7(1)
	PDF	*164.2(2)*	*164.2(2)*	*163.9(2)*	*164.7(2)*
Si-O3 (2x)	Bragg	162.0(1)	162.0(1)	161.3(1)	161.9(1)
	PDF	*163.8(2)*	*163.9(2)*	*165.2(2)*	*164.3(2)*
<Si-O>	Bragg	162.6(1)	162.6(1)	160.7(1)	162.4(1)
	PDF	*163.3(2)*	*163.5(2)*	*163.5(3)*	*163.8(2)*
BVS	Bragg	3.99(1)	3.99(2)	4.19(2)	4.00(1)
	PDF	*3.89(2)*	*3.87(2)*	*3.84(3)*	*3.84(1)*
Mg(1)-O(1) (2x)	Bragg	208.5(1)	208.5(1)	210.5(1)	208.4(1)
	PDF	*208.3(1)*	*208.5(2)*	*210.7(8)*	*208.7(2)*
Mg(1)-O(2) (2x)	Bragg	207.2(1)	207.2(1)	207.2(1)	207.2(1)
	PDF	*207.7(1)*	*208.3(1)*	*208.1(7)*	*207.6(2)*
Mg(1)-O(3) (2x)	Bragg	213.3(1)	213.3(1)	214.0(1)	213.3(1)
	PDF	*212.7(2)*	*212.9(2)*	*214.8(8)*	*212.7(2)*
	Bragg	209.7(1)	209.7(1)	210.6(1)	209.6(1)

<Mg(1)-O>	*PDF*	*209.6(2)*	*209.9(2)*	*211.2(8)*	*209.7(2)*
BVS	Bragg	2.02(1)	2.02(1)	1.97(1)	2.02(1)
	PDF	*2.02(1)*	*2.00(1)*	*1.94(4)*	*2.02(1)*
Mg(2)-O(1)	Bragg	217.1(1)	217.0(1)	217.8(1)	216.9(1)
	PDF	*217.5(3)*	*216.8(4)*	*218.2(4)*	*216.6(2)*
Mg(2)-O(2)	Bragg	206.2(1)	206.1(1)	206.9(1)	206.1(1)
	PDF	*204.8(3)*	*204.5(3)*	*203.0(4)*	*204.7(2)*
Mg(2)-O(3) (2x)	Bragg	208.2(1)	208.2(1)	208.6(1)	208.1(1)
	PDF	*206.3(2)*	*206.4(3)*	*205.7(5)*	*206.4(2)*
Mg(2)-O(3) (2x)	Bragg	220.4(1)	220.4(1)	220.6(1)	220.4(1)
	PDF	*222.5(3)*	*222.4(4)*	*220.5(4)*	*221.9(2)*
<Mg(2)-O>	Bragg	213.4(1)	213.4(1)	213.9(1)	213.3(1)
	PDF	*213.3(3)*	*213.2(4)*	*212.3(5)*	*213.0(2)*
BVS	Bragg	1.85(1)	1.85(1)	1.82(1)	1.85(1)
	PDF	*1.86(2)*	*1.87(2)*	*1.92(2)*	*1.88(1)*

Table S4 Defective structure candidates along with their symmetry analysis, formation energy, and R_{PDF} values. GOSWD = Geometry Optimized Structure Without Defect, F = Frenkel, I = Interstitial, V = Vacancy. Several structure models (noted with *) fall back to the pristine structure upon optimization.

Defective structure candidate	Unit cell size	Space group	Formation energy /eV	R_{PDF} /%
GOSWD	1	*Pbnm* (62)*	0.01	23
GOSWD	*2x2x2*	*Pbnm* (62)*	*0.01*	*27*
		**Frenkel defect**		
Mg(1)	1	$P\bar{1}$ (2)	2.98	25
Mg(1)	*2x2x2*	*P*1 (1)	*5.41*	*20*
Mg(2)	1	*Pbnm* (62)*	0.01	26
Mg(2)	*2x2x2*	*Pbnm* (62)*	*0.01*	*20*
O(1)	1	*P*1 (1)	7.92	23
O(1)	*2x2x2*	*P*1 (1)	*7.64*	*21*
O(2)	1	*Pbnm* (62)*	0.01	27
O(2)	*2x2x2*	*P*1 (1)	*7.69*	*19*
O(3)	1	*Pbnm* (62)*	0.01	27
O(3)	*2x2x2*	*P*1 (1)	*7.69*	*19*
		**Vacancy defect**		
Mg(1)	1	*P*1 (1)	9.06	26

Mg(1)	*2x2x2*	$P1$ (1)	*9.11*	*19*
Mg(2)	1	Pm (6)	10.14	23
Mg(2)	*2x2x2*	Pm (6)	*10.27*	*19*
O(1)	1	Pm (6)	6.44	23
O(1)	*2x2x2*	Pm (6)	*6.51*	*22*
O(2)	1	$P1$ (1)	6.54	22
O(2)	*2x2x2*	$P1$ (1)	*6.56*	*21*
O(3)	1	$P1$ (1)	6.54	23
O(3)	*2x2x2*	$P1$ (1)	*6.56*	*19*
Si	1	$P1$ (1)	12.37	25
Si	*2x2x2*	$P1$ (1)	*12.37*	*19*
**Schottky defect**				
Mg(1)-O(1)	1	$P1$ (1)	10.59	25
Mg(1)-O(1)	*2x2x2*	$P1$ (1)	*11.64*	*19*
Mg(1)-O(2)	1	$P1$ (1)	8.79	28
Mg(1)-O(2)	*2x2x2*	$P1$ (1)	*9.88*	*19*
Mg(1)-O(3)	1	$P1$ (1)	9.22	28
Mg(1)-O(3)	*2x2x2*	$P1$ (1)	*9.80*	*20*
Mg(2)-O(1)	1	$P1$ (1)	9.69	24
Mg(2)-O(1)	*2x2x2*	$P1$ (1)	*13.99*	*20*
Mg(2)-O(2)	1	$P1$ (1)	9.55	28
Mg(2)-O(2)	*2x2x2*	$P1$ (1)	*10.87*	*19*
Mg(2)-O(3)	1	$P1$ (1)	9.55	28
Mg(2)-O(3)	*2x2x2*	$P1$ (1)	*10.98*	*23*
**Charge defect**				
Mg I +0	2x1x2	$P1$ (1)	3.73	27
Mg I +2	*2x1x2*	$P1$ (1)	*-5.25*	*27*
Mg V -2	2x1x2	$P1$ (1)	9.05	25
Mg V +0	*2x1x2*	$P1$ (1)	*9.25*	*25*
O I +0	2x1x2	$P1$ (1)	1.37	27
O I -2	*2x1x2*	$P1$ (1)	*10.24*	*23*
O V +2	2x1x2	$P1$ (1)	1.49	27
O V +0	*2x1x2*	$P1$ (1)	*6.66*	*27*
Si I +0	2x1x2	$P1$ (1)	6.39	27
Si I +4	*2x1x2*	$P1$ (1)	*-9.07*	*28*

Table S5 Formation energy of charge defect structures computed using different fixed supercell size and dilute limit*. F = Frenkel, I = Interstitial, V = Vacancy.

**Defective structure candidate**	**Formation energy /eV Unit cell size**					
	**1**	**2x1x2**	**3x1x2**	**3x2x2**	**3x2x3**	**Dilute limit**
Mg I +0	3.13	3.73	3.99	4.29	4.24	
Mg I +2	*-6.45*	*-5.25*	*-4.99*	*-4.78*	*-4.90*	*-4.64*
Mg V +0	9.15	9.25	9.27	9.38	11.49	
Mg V -2	*6.46*	*9.05*	*9.38*	*9.80*	*9.77*	*10.21*
O I +0	1.76	1.37	1.38	1.40	1.24	
O I -2	*1.74*	*10.24*	*10.53*	*6.32*	*6.46*	*-9.63*
O V +0	6.45	6.66	6.73	6.72	6.56	
O V +2	*0.77*	*1.49*	*1.65*	*1.87*	*1.71*	*2.00*
Si I +0	4.33	6.39	8.67	6.77	6.68	
Si I +4	*-13.39*	*-9.07*	*-8.14*	*-7.30*	*-7.13*	*-4.92*
